# Postprandial Metabolomic Profiling: Insights into Macronutrient-Specific Metabolic Responses in Healthy Individuals

**DOI:** 10.3390/nu16213783

**Published:** 2024-11-04

**Authors:** Awad Alshahrani, Shereen M. Aleidi, Mohammed Al Dubayee, Reem AlMalki, Rajaa Sebaa, Mahmoud Zhra, Anas M. Abdel Rahman, Ahmad Aljada

**Affiliations:** 1 College of Medicine, King Saud Bin Abdulaziz University for Health Sciences, King Abdullah International Medical Research Center, Ministry of National Guard Health Affairs (MNG-HA), Riyadh 11426, Saudi Arabia; shahranias@mngha.med.sa (A.A.); aldubayeemo@ngha.med.sa (M.A.D.); 2 Department of Biopharmaceutics and Clinical Pharmacy, School of Pharmacy, The University of Jordan, Amman 11942, Jordan; s.aleidi@ju.edu.jo; 3 College of Pharmacy, University of Sharjah, Sharjah 27272, United Arab Emirates; 4 Metabolomics Section, Department of Clinical Genomics, Center for Genomics Medicine, King Faisal Specialist Hospital and Research Centre (KFSHRC), Riyadh 11211, Saudi Arabia; rgalmalki@kfshrc.edu.sa; 5 Department of Medical Laboratories, College of Applied Medical Sciences, Shaqra University, Shaqra 11961, Saudi Arabia; r.sebaa@su.edu.sa; 6 Department of Biochemistry and Molecular Medicine, College of Medicine, Alfaisal University, Riyadh 11533, Saudi Arabia; mzahra@alfaisal.edu

**Keywords:** macronutrients, untargeted metabolomics profiling, lipid metabolism, 3’-O-Methylguanosine, insulin sensitivity, postprandial response

## Abstract

Background/Objectives: Understanding the metabolic responses to different macronutrients is crucial for assessing their impacts on health. This study aims to investigate the postprandial metabolomic profiles of healthy individuals following the consumption of glucose, protein, and lipids. Methods: Twenty-three healthy, normal-weight adults participated in the study, randomly assigned to consume 300 kcal from glucose, protein, or lipids after an overnight fast. Blood samples were collected at baseline and at 1, 2, and 3 h post-ingestion. An untargeted metabolomic approach using mass spectrometry was employed to analyze plasma metabolites. Results: In total, 21, 59, and 156 dysregulated metabolites were identified after glucose, protein, and lipid intake, respectively. Notably, 3’-O-methylguanosine levels decreased significantly after glucose consumption while remaining stable during lipid intake before increasing at 2 h. Common metabolites shared between glucose and lipid groups included 3’-O-methylguanosine, 3-oxotetradecanoic acid, poly-g-D-glutamate, and triglyceride (TG) (15:0/18:4/18:1). Conclusions: The findings highlight distinct metabolic responses to macronutrient intake, emphasizing the role of specific metabolites in regulating postprandial metabolism. These insights contribute to understanding how dietary components influence metabolic health and insulin sensitivity.

## 1. Introduction

Macronutrients, encompassing carbohydrates, lipids, and proteins, are nutrients that the body needs in large amounts and are crucial to supplying the body with energy and essential components for various tissues [1,2]. They exert distinct metabolic effects on both health and energy balance [3]. For example, carbohydrate consumption elevates blood glucose levels and triggers insulin secretion, facilitating glucose absorption by tissues and storing it as glycogen [4]. Lipids, or dietary fats, are the most energy-rich macronutrients and have important roles in the body, including storing energy as body fat, maintaining cellular structure, producing sex hormones, facilitating the absorption of fat-soluble vitamins, and regulating body temperature [5]. Proteins are large molecules composed of varying amounts and combinations of amino acids. They are a less efficient energy source than carbohydrates or lipids [6]. However, consuming dietary proteins provides the body with essential amino acids that play a key role in synthesizing enzymes, hormones, antibodies, cytokines, transporters, and neurotransmitters and inhibiting protein catabolism [5,6].

The maintenance of metabolic health relies on achieving a balance between caloric intake and expenditure. However, it is widely recognized that macronutrients elicit different metabolic responses [7], which can be influenced by age, gender, genetics, and body composition [7]. In recent years, there has been growing interest in postprandial metabolism owing to its association with dynamic metabolic responses during the post-meal phase and overall health [8]. Furthermore, assessing the postprandial metabolic response to dietary challenges can offer valuable insight into metabolic health and the prediction of diseases, surpassing the measurement of fasting levels of blood glucose or triglycerides [8]. Each meal is comprised of micronutrients that trigger complex and timely changes within the body, transitioning from the fasting or pre-meal state to the post-meal state. These changes encompass the regulation of metabolic pathways and result in modifications in the concentrations of various metabolites in the blood circulation [8].

Metabolomics is a comprehensive approach based on the high-throughput measurement and characterization of small-molecule metabolites in a biological sample. It is a highly sensitive method and offers a more holistic view of metabolic changes. It can identify even minor changes in metabolite levels, facilitating a more precise metabolic health assessment. Its application in the nutrition era provides a robust platform to characterize the metabolic responses to dietary challenges [7,9]. Furthermore, it enables exploration of the patterns of time-based changes in endogenous and dietary-derived metabolite levels and the assessment of individual variations in baseline metabolic health and/or disease status [7,9]. Several human nutritional studies have examined the metabolic response to dietary challenges utilizing mixed meals [7,10,11] or standardized challenge tests such as the oral glucose tolerance test (OGTT) [8,12,13] and oral lipid tolerance test (OLTT) [8,13]. However, variations in the experimental design, analytical protocol, diet intervention period, and evaluation of the systemic metabolic response in these studies present opportunities for further research with enhanced methodologies to deepen our understanding of the nutrient metabolome.

This study uses an untargeted metabolomic approach to analyze the plasma metabolomic profiles of healthy lean individuals after consuming glucose, protein, or lipids for 3 h. It examines the changes in metabolite levels before and after caloric intake, shedding light on metabolic alterations post-macronutrient consumption. The results improve our understanding of glucose, protein, and lipid metabolism by identifying related downstream metabolites and pathways. In addition, the study aims to identify potential biomarkers for assessing metabolic health and disease status. Studying time-dependent changes in metabolite levels offers insight into the dynamic nature of nutrient metabolism and individual variability.

## 2. Methods

### 2.1. Study Population and Setting

The determination of the sample size for this study was conducted through a power analysis, utilizing data derived from prior investigations in postprandial metabolomics. Previous studies, including those by LaBarre et al. (2021) and Weinisch et al. (2022), have indicated notable metabolic alterations following meals, with sample sizes between 15 and 30 participants in healthy cohorts [7,8,11,12]. Our power analysis, based on the anticipated effect sizes for metabolite fluctuations identified in these earlier works, indicated that a sample size of 23 participants would adequately allow for the detection of significant postprandial responses at an 80% power threshold. Although increasing the sample size could improve the generalizability of our results, the scale of our study aligned well with existing postprandial metabolomics research and was deemed adequate for the untargeted methodology employed in this investigation.

A total of 23 (*n* = 23) healthy adult volunteers were included, and they were randomly divided into three groups. Individuals in each group were provided with an equal number of calories (300 kcal) from either glucose, protein, or lipids following an overnight fast. The first group comprised 7 volunteers who were given glucose (NERL Trutol 75 g, Thermo Fisher Scientific, Waltham, MA, USA). The second group consisted of 10 volunteers who received protein (Isopure unflavored Whey Protein Isolate (WPI) powder, Glanbia Nutritionals, Fitchburg, WI, USA), containing 26 g per serving of 100% WPI, free of fat, carbohydrates, fillers, sugars, and lactose. The third group included 6 volunteers who were administered lipids (90 g whipping cream, 31.5 g fat, 1.7 g protein, and 2.25 g carbohydrates, Almarai, Riyadh, Saudi Arabia). The lipid source utilized in this study was commercially available whipping cream, which contains a small amount of protein (1.7 g) and carbohydrates (2.25 g); this choice was made due to the difficulty of obtaining high-lipid preparations without any protein or carbohydrates that would also be acceptable to participants. The cream and protein mixtures were diluted with water to form 300 mL solutions. The specified duration for consumption was set at 5 min for the 300 kcal meals. Each participant had four blood samples collected: one at baseline or zero time (before caloric intake) and three after caloric intake at various time points (1, 2, and 3 h into the postprandial phase). The number of blood samples collected for each group was as follows: glucose group, *n* = 28; protein group, *n* = 40; and lipid group, *n* = 24. The biochemical parameters measured to assess kidney function included serum creatinine and blood urea nitrogen, while liver function was evaluated using alanine aminotransferase and aspartate aminotransferase. Lipid profiles were assessed through total cholesterol and triglycerides. All participants had normal liver and kidney function test results, possessed typical lipid profiles, and were not taking any medications. Blood samples were collected using Na-EDTA as an anticoagulant, the tubes were centrifuged at 1000–2000× *g* for 10 min in a refrigerated centrifuge, and then plasma was obtained.

### 2.2. Ethics Statement

All procedures conducted in this study adhered to the ethical standards outlined in the Declaration of Helsinki and the guidelines set forth by the Universal International Conference on Harmonization–Good Clinical Practice (ICH-GCP). Before their participation, participants were required to provide written informed consent indicating their approval of involvement in the study. The Institutional Review Board (IRB) of the Ministry of National Guard Health Affairs (MNGHA) reviewed and approved the study protocol (approval no. SP17/380/R).

### 2.3. Glucose, Insulin, and BMI Measurements

Whole blood glucose concentrations were assessed utilizing the ACCU Check Active Blood Glucose Monitor. Insulin levels were quantified employing the ALPCO insulin ELISA kit (Salem, NH, USA), following the guidelines provided by the manufacturer. Subsequently, the body mass index (BMI) of each participant was computed using the formula: weight in kilograms (kg) divided by the square of height in meters (m).

### 2.4. Sample Preparation

Metabolites were isolated from plasma following the methodology described in a previous study [14]. In summary, 50 μL of plasma sample was combined with 950 μL of a (1:1) (*v*/*v*) extraction solvent mixture of acetonitrile (ACN) and methanol (MeOH). Pooled plasma quality control (QC) samples were also prepared using portions from all study samples to ensure system stability and suitability. The mixtures were agitated on a thermomixer at 600 rpm at room temperature (RT) for one hour (Eppendorf, Hamburg, Germany). Subsequently, the samples underwent centrifugation at 16,000 rpm at 4 °C for 10 min. The sample supernatant was transferred to a new Eppendorf tube and completely evaporated using a SpeedVac (Christ, Germany). The dried sample was reconstituted in 100 μL of mobile phases A:B (1:1) (A: 0.1% formic acid in dH_2_O, B: 0.1% formic acid in a 1:1 (*v*/*v*) mixture of ACN:MeOH).

### 2.5. Metabolomic Analysis

The metabolomic profile for each sample was generated using the Waters Acquity UPLC system in conjunction with a Xevo G2-S QTOF mass spectrometer, which was equipped with an electrospray ionization source (ESI), as detailed in a previous study [14]. The extracted metabolites were chromatographed using an ACQUITY UPLC system with an XSelect (100 × 2.1 mm, 2.5 μm) column (Waters Ltd., Elstree, UK). The mobile phase combined 0.1% formic acid in dH_2_O as solvent A and 0.1% formic acid in (1:1) (*v*/*v*) ACN:MeOH as solvent B. A gradient elution program was executed with the following parameters: 0–16 min 95–5% A, 16–19 min 5% A, 19–20 min 5–95% A, and 20–22 min 5–95% A, at a flow rate of 300 µL/min. Mass spectrometry spectra were obtained under positive and negative electrospray ionization modes (ESI+, ESI−). The MS conditions included a source temperature of 150 °C, a desolvation temperature of 500 °C for both ionization modes, a capillary voltage of 3.20 kV (ESI+) or 3 kV (ESI−), a cone voltage of 40 V, desolvation gas flow of 800.0 L/h, and cone gas flow of 50 L/h. The collision energies for low and high functions were set at off and 10 V to 50 V, respectively, in MSE mode. The mass spectrometer was calibrated using sodium formate in the range of 100–1200 Da for both ionization modes. Additionally, leucine-enkephalin was used as a lock mass compound (external reference for the ion *m*/*z* 556.2771 in ESI+ and 554.2615 in ESI−) and was infused continuously, alternating between the sample and reference every 45 and 60 s for ESI+ and ESI-, respectively. Data were acquired in continuum mode using Masslynx™ V4.1 workstation (Waters Inc., Milford, MA, USA). Quality control samples (QCs) were run by combining 10 µL from every study sample, which were extracted and then introduced to the instrument with randomization to confirm the system’s stability. Subsequently, they were analyzed according to the standard protocol in our lab. The criteria for acceptance included ensuring that all QC samples were distinct from the other study groups, and when grouped had relative standard deviations (RSD%) of less than 40%.

### 2.6. Data and Statistical Analysis

The MS raw data were processed following standard processing steps, which included alignment based on the *m*/*z* value and the retention time of ion signals, peak picking, and signal filtering based on peak quality. This processing was performed using Progenesis QI v.3.0 software from Waters Technologies (Milford, MA, USA). To perform multivariate statistical analysis, MetaboAnalyst version 5.0 from McGill University (Montreal, Canada) was utilized (http://www.metaboanalyst.ca, accessed on 21 April 2023) [15]. The datasets comprising compound names and their raw abundances were subjected to median normalization, Pareto scaling, and log transformation to maintain their normal distribution.

To address individual differences prior to macronutrient intake, baseline metabolite levels were measured and used as reference points for postprandial comparisons. Postprandial metabolite levels were normalized to each participant’s baseline values, which helped to reduce the influence of pre-existing variability across individuals. By doing so, we ensured that observed metabolic changes primarily reflected the impact of macronutrient intake rather than individual differences. Additionally, we applied robust multivariate statistical methods, including partial least squares-discriminant analysis (PLS-DA) and orthogonal partial least squares-discriminant analysis (OPLS-DA). These techniques are highly effective for handling complex datasets, which allowed us to account for individual variability while focusing on group-level metabolic responses. This approach allowed us to capture the true metabolic effects of macronutrient intake with minimized bias from baseline differences.

The normalized datasets were subsequently used to construct PLS-DA and OPLS-DA models. The fitness of the OPLS-DA models, indicated by R2Y values, and their predictive ability (Q2) were evaluated through permutation validation using 100 samples [16]. For univariate analysis, we utilized Mass Profiler Professional (MPP) v.15.0 software from Agilent (Santa Clara, CA, USA). One-way analysis of variance (ANOVA) followed by Tukey’s post-hoc test was performed on the three groups, identifying significant features with a *p*-value of less than 0.05. Venn diagrams were generated using MPP v.15.0 software to visualize shared and unique metabolites between the macronutrient groups [17,18,19]. Heatmap analysis, based on the Pearson distance measure and Pearson similarity test, was performed to visualize altered features. Finally, pathway analysis was conducted on significantly dysregulated endogenous metabolites to identify impacted metabolic pathways.

### 2.7. Metabolites Identification

Progenesis QI software was utilized to select and tag the significant features in each dataset for peak alignment and molecular annotation. Their accurate precursor masses were obtained to identify the chemical structures of metabolites, and theoretical MS/MS fragmentation tolerance values were established. A 5 ppm mass window was set for the Human Metabolome Database (HMDB) while METLIN MS/MS (www.metlin.scripps.edu; accessed on 20 March 2023), employing a 5 ppm fragmentation filter, which could be either in silico or empirical. Exogenous compounds such as drugs, food additives, and environmental compounds were manually excluded from the final list to ensure accuracy.

## 3. Results

### 3.1. The Clinical Features and Demographic Profile of the Participants in the Study

Table 1 presents the demographic data and clinical characteristics of the study population at baseline and plasma glucose and insulin concentrations following caloric intake. The results are presented as the mean ± SEM; *: *p* < 0.05. Most of the participants were males matched in age and BMI. Insulin levels were significantly increased after 1 h of glucose and protein intake (42.2 ± 18.80, 28.483 ± 4.048 µU/mL, respectively) (*p* < 0.05). After 1 h of lipid intake, the increase in insulin levels was not significant. It should be noted that only one female participant was included in the glucose group. As a sensitivity analysis, we re-ran the analysis excluding the data from this participant. The results remained unchanged, confirming that the inclusion of this participant did not influence the overall outcomes. Nevertheless, we recognize the importance of having a more balanced gender distribution in future studies.

### 3.2. The Comprehensive Metabolomic Analysis and Contrasts Among the Study Cohorts

**Figure 1:**
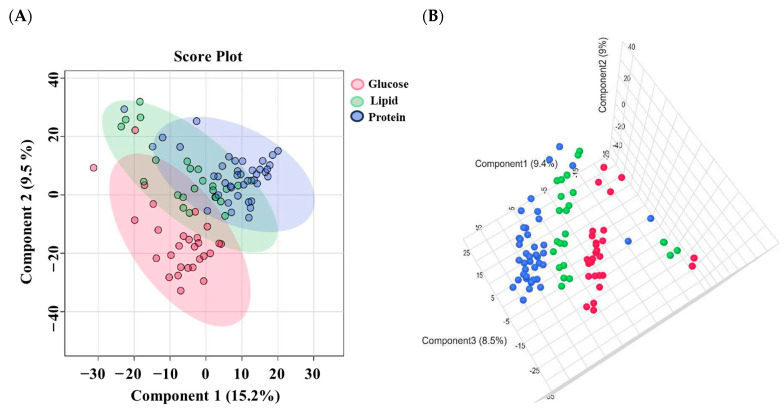
PLS-DA model between the three study groups. (**A**) 2D model and (**B**) 3D model. The PLS-DA model demonstrates a clear distinction among the three study groups. It reveals a noticeable segregation between the glucose and protein groups, a subtle differentiation between the glucose and lipid groups. Nevertheless, there exists an intersection between the lipid and protein group.

A total of 44,012 mass ion features were detected, 23,289 in positive and 20,723 in negative ionization modes. The features used for analysis were 23,238 in the glucose group, 23,981 in the protein group, and 26,611 in the lipid group. The metabolomic profiles of the healthy participants following the consumption of equicaloric amounts of macronutrients (glucose, protein, and lipids) were analyzed using the partial least squares discriminant analysis (PLS-DA) model (Figure 1). This model was employed to explore the clustering and differentiation among the various groups. Figure 1 illustrates the partial separation in the metabolic profiles between the glucose and protein groups and the slight separation between the glucose and lipid groups. However, no distinct separation was observed between the lipid and protein groups.

**Figure 2:**
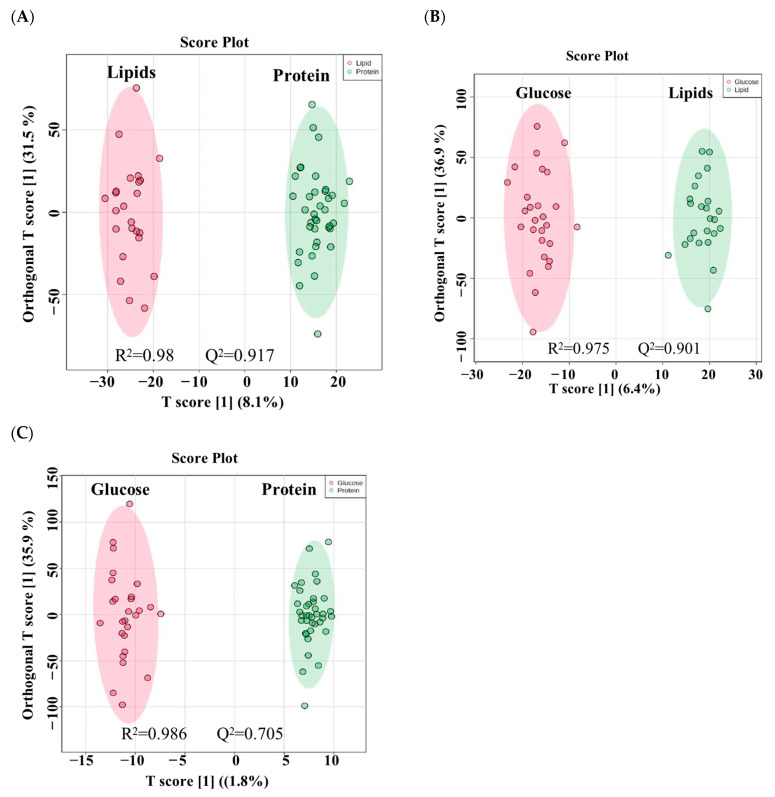
OPLS-DA model of every two groups in the study. (**A**) Separation between lipid and protein groups, R^2^ = 0.98 and Q^2^ = 0.917. (**B**) Separation between glucose and lipid groups, R^2^ = 0.975 and Q^2^ = 0.901. (**C**) Separation between glucose and protein groups, R^2^ = 0.986 and Q^2^ = 0.705. The robustness of the created models was evaluated by the fitness of the model (R^2^Y) and predictive ability (Q^2^) values in a larger dataset (*n* = 100).

Furthermore, an OPLS-DA model was utilized to conduct binary comparisons between each pair of groups, visualizing the classification effect, clustering, and separation in the plot. The model demonstrated a clear and significant separation between the groups, with evident distinctions between the lipid and protein groups (R^2^ = 0.98 and Q^2^ = 0.917, Figure 2A), glucose and lipid groups (R^2^ = 0.975 and Q^2^ = 0.901, Figure 2B), and glucose and protein groups (R^2^ = 0.986 and Q^2^ = 0.705, Figure 2C), indicating variations in metabolite expression following the intake of different macronutrients.

### 3.3. The Postprandial Phase Elicits Distinct Plasma Metabolome Responses to Each Macronutrient

**Figure 3:**
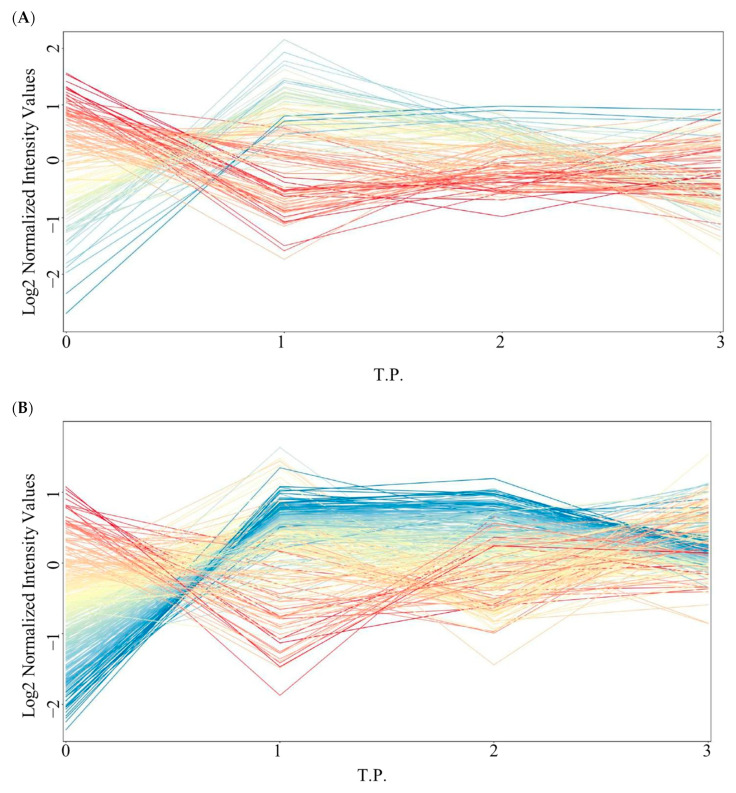

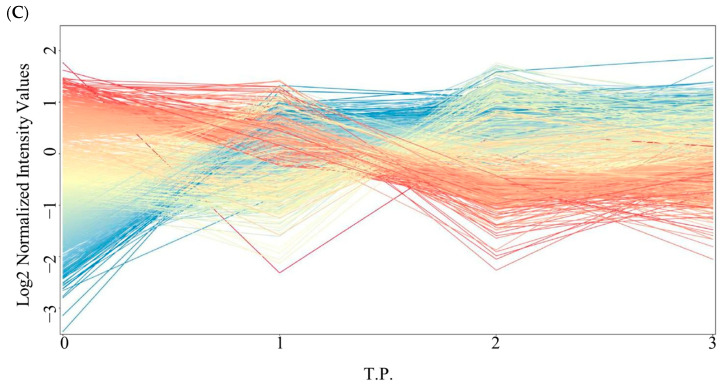
Postprandial plasma metabolomic profiles following the consumption of different macronutrients (glucose, protein, and lipids) at various time intervals (0, 1, 2, and 3 h). (**A**) Profile of 130 significant metabolites that were dysregulated in the glucose group during the time points (0, 1, 2, and 3) (Tukey’s post-hoc, *p* < 0.05). (**B**) Profile of 316 significant metabolites that were dysregulated in the protein group during the time points (0, 1, 2, and 3) (Tukey’s post-hoc, *p* < 0.05). (**C**) Profile of 1324 significant metabolites that were dysregulated in the lipid group during the time points (0, 1, 2, and 3) (Tukey’s post-hoc, *p* < 0.05). The red color represents downregulated, blue color represents upregulated, and yellow represents unsignificant.

An investigation was conducted to determine the metabolites that exhibited significant changes following the consumption of different macronutrients (glucose, protein, and lipids). One-way ANOVA analysis was performed for each group at various time intervals (0, 1, 2, and 3 h) with a significance level set at *p* ≤ 0.05. In the glucose group, 130 metabolites showed significant dysregulation across multiple time points (Figure 3A). Looking at the metabolomic profiles of these metabolites, the most significant changes in their levels (either up or downregulation) occurred after 1 h of glucose intake (Figure 3A). Upon excluding exogenous metabolites, such as drug metabolites or any metabolites that were not related to dietary-derived metabolites, only 21 metabolites were identified as human endogenous metabolites (glucose-associated metabolites, *n* = 21).

**Figure 4:**
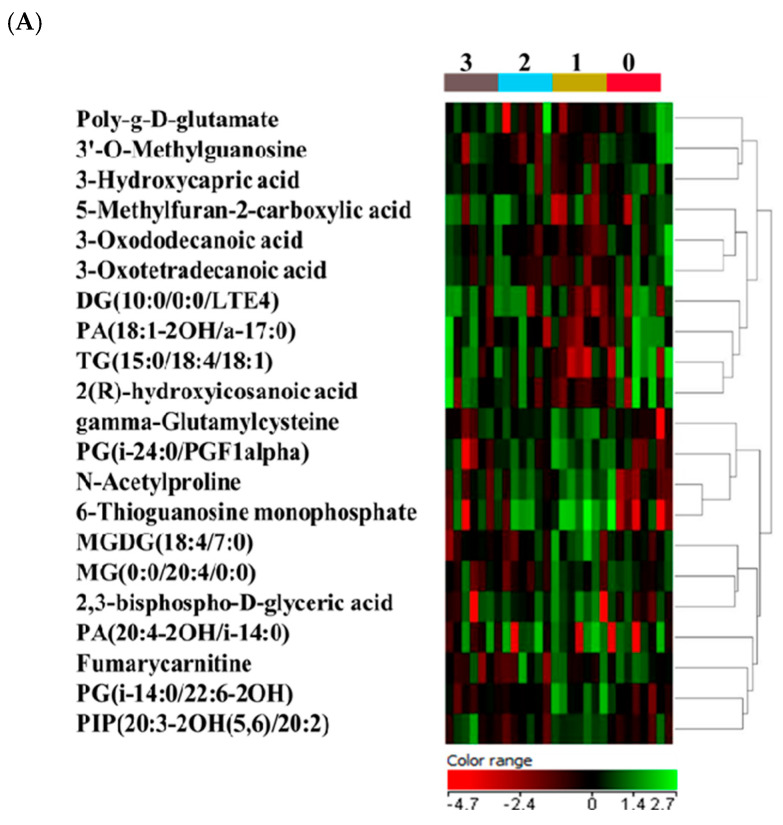

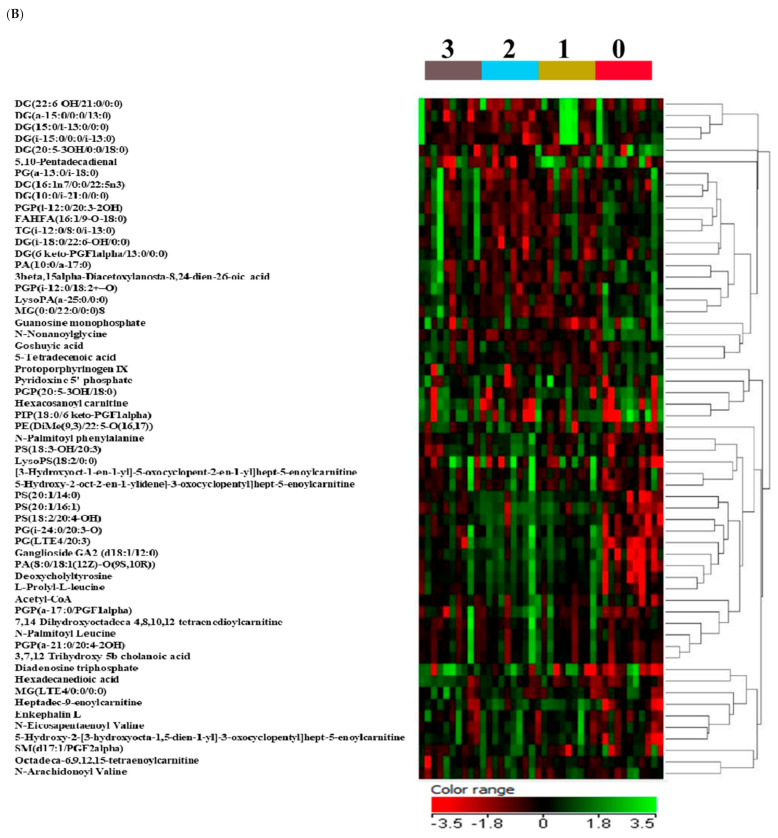

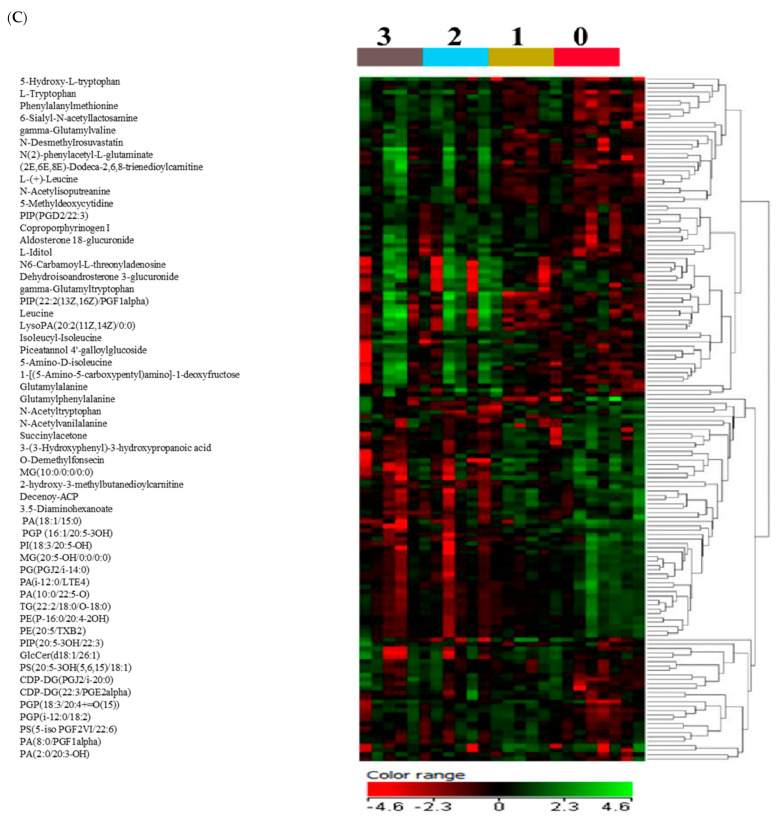
Heatmaps of the dysregulated human endogenous metabolites after macronutrient intake at different time points. (**A**) Heatmap of 21 dysregulated endogenous metabolites related to glucose intake. (**B**) Heatmap of 59 dysregulated endogenous metabolites related to protein intake. (**C**) Heatmap of 156 dysregulated endogenous metabolites related to lipid intake.

Similarly, the protein and lipid groups displayed 316 and 1324 significantly dysregulated metabolites at multiple time points, respectively (Figure 3B,C). The metabolomic profiles of metabolites were significantly changed after 1 h, followed by a slight change at 2 h after protein intake (Figure 3B). After lipid intake, the metabolomic changes occurred after both 1 and 2 h. However, it was more prominent after 2 h (Figure 3C), indicating the time changes that occurred in plasma metabolite levels after macronutrient intake varied. Within these significant metabolites, only 59 and 156 were recognized as endogenous to humans (protein-associated metabolites, *n* = 59; lipid-associated metabolites, *n* = 156). For each macronutrient-related metabolite, one-way ANOVA analysis was performed at various time intervals (0, 1, 2, and 3 h) with a significance level set at *p* ≤ 0.05. The changes in the levels of these metabolites were detected, and the specific metabolites that were dysregulated were identified (Figure 4A–C). The details are presented in Supplementary Tables S1–S3.

Analysis of the metabolic pathways impacted by the significantly dysregulated human endogenous metabolites following macronutrient intake indicated a notable association with amino acid metabolism. Specifically, pathways involving phenylalanine, tyrosine, and tryptophan biosynthesis and metabolism were identified as the most relevant.

### 3.4. Common Dysregulated Endogenous Metabolites Between the Three Groups of Macronutrients

**Figure 5:**
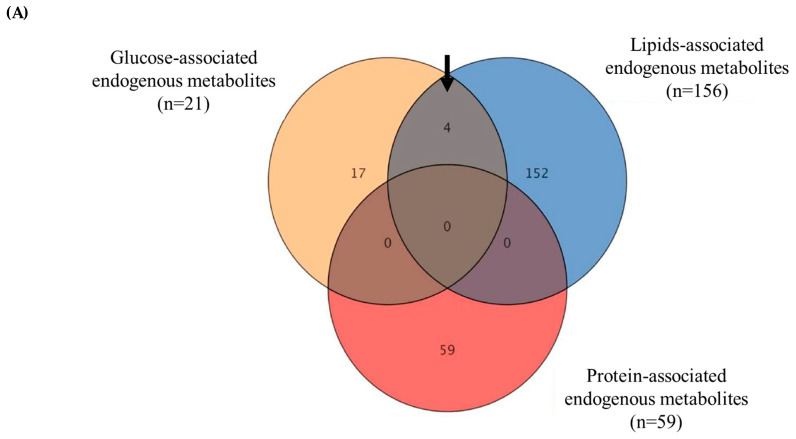

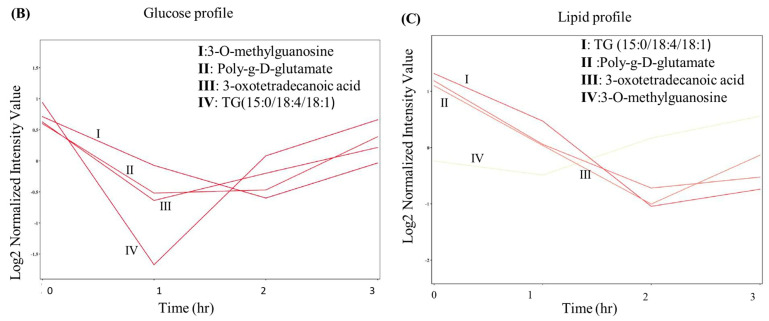
Commonly dysregulated metabolites among the three macronutrient groups at different time points. (**A**) Venn diagram illustrating the overlap between the endogenous metabolites associated with each macronutrient (glucose-associated metabolites, *n* = 21; lipid-associated metabolites, *n* = 156; and protein-associated metabolites, *n* = 59) using one-way ANOVA (cut-off no correction *p*-value ≤ 0.05). (**B**,**C**) Metabolomic profiles of the 4 identified dysregulated metabolites common between the glucose (**B**) and lipid (**C**) groups during the different time points (0, 1, 2, and 3 h).

The endogenous metabolites linked with each macronutrient (glucose-associated metabolites, *n* = 21; protein-associated metabolites, *n* = 59; and lipid-associated metabolites, *n* = 156) were overlapped through Venn diagram analysis (Figure 5A). A one-way ANOVA analysis and setting a cut-off *p* < 0.05 for the three macronutrient groups across multiple time points found that four endogenous metabolites were shared between glucose- and lipids-associated metabolites (Figure 5A). These metabolites consisted of 3′-O-methylguanosine, 3-oxotetradecanoic acid, poly-g-D-glutamate, and triglyceride (TG) (15:0/18:4/18:1). Interestingly, there were no common metabolites identified between glucose- and protein- or lipid- and protein-associated metabolites.

The metabolomic profiling of the four identified common dysregulated metabolites is depicted in the glucose and lipids groups at various time points during the postprandial phase (0, 1, 2, and 3 h) (Figure 5B,C). Following glucose consumption, the plasma concentrations of the four metabolites exhibited varying degrees of decrease before rising again at multiple time points. For instance, one hour after glucose ingestion, the levels of poly-g-D-glutamate, 3-oxotetradecanoic acid, and TG (15:0/18:4/18:1) decreased significantly, with TG (15:0/18:4/18:1) showing the most pronounced decline (Figure 5B). Conversely, after two hours, the level of 3′-O-methylguanosine reached its lowest point, while the level of TG (15:0/18:4/18:1) notably increased (Figure 5B). In the context of lipid intake, the plasma levels of TG (15:0/18:4/18:1), poly-g-D-glutamate, and 3-oxotetradecanoic acid began to decrease and hit their nadir after two hours of lipid consumption, suggesting a slower metabolic turnover of these metabolites following lipid intake compared to glucose intake.

**Figure 6:**
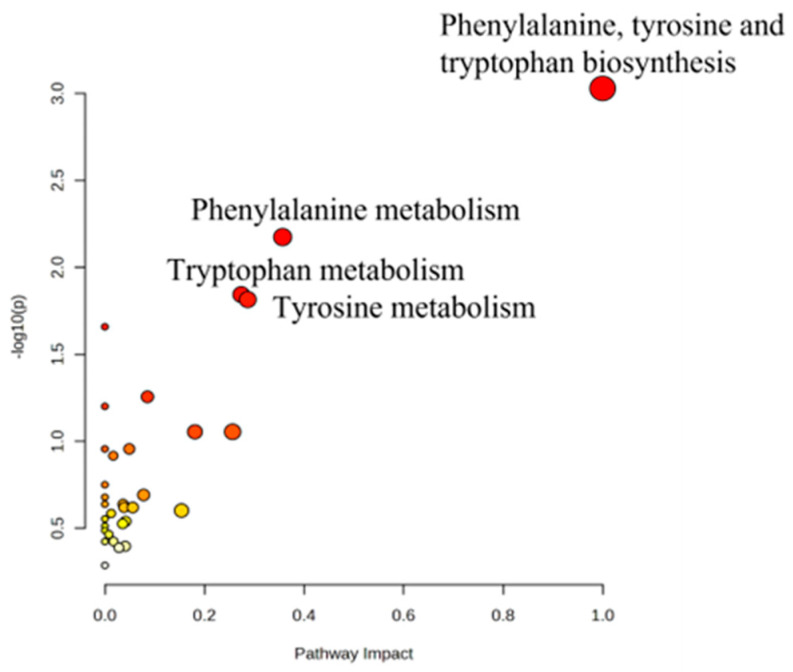
Pathway analysis of the significant metabolites dysregulated in the three macronutrient groups. The color variation (yellow to red) shows the different significance levels of metabolites in the data.

Moreover, analysis of the metabolic pathways impacted by the significantly dysregulated human endogenous metabolites following macronutrient intake indicated a notable association with amino acid metabolism. Specifically, pathways involving phenylalanine, tyrosine, and tryptophan biosynthesis and metabolism were identified as the most relevant (Figure 6).

## 4. Discussion

Metabolomics, a powerful tool in nutritional research, has been used to identify biomarkers of dietary intake, including macronutrients [20,21]. These biomarkers can reflect habitual dietary patterns, such as red meat and vegetable intake [22]. Despite these advancements, metabolomics in nutritional epidemiology is still relatively new, with the potential to uncover diet–disease associations in large cohort studies [23]. In this study, the changes in metabolites after the consumption of equicaloric amounts (300 kcal) of glucose, whey protein, or lipids (whipping cream) by healthy individuals were investigated. The results revealed distinct clustering of metabolite expression within each macronutrient group using the OPLS-DA model. Specifically, glucose consumption led to dysregulation of 21 metabolites, whey protein intake resulted in dysregulation of 59 metabolites, and lipid intake caused dysregulation of 156 metabolites. Notably, the consumption of pure glucose or protein induced pronounced insulin-dependent alterations. In comparison, lipid intake led to complex changes. Further analysis using a Venn diagram identified four common dysregulated metabolites between the glucose and lipid groups, namely 3′-O-methylguanosine (3′-OMG), 3-oxotetradecanoic acid (3-OTDA), poly-g-D-glutamate, and triglyceride (TG) (15:0/18:4/18:1). However, no common dysregulated metabolites were found between the protein and glucose or protein and lipid groups.

Several carnitine derivatives, including hydroxy propionyl carnitine and L-acetylcarnitine, nona-5,7-dienedioylcarnitine, hexacosanoyl carnitine, and fumarycarnitine were among the dysregulated metabolites after consumption of the three groups of macronutrients. Carnitine and its derivatives are crucial in regulating intracellular glucose and lipid metabolism [24]. The increase in L-acetylcarnitine levels post-lipid intake suggested a shift toward β-oxidation for energy production [24]. Previous research has indicated that L-carnitine supplementation may benefit individuals with conditions like insulin resistance, diabetes mellitus, and dyslipidemia [25]. Furthermore, a study measuring plasma acetylcarnitine levels in response to a high-fat challenge in overweight and obese individuals demonstrated a decrease in unsaturated acetylcarnitine species over 6 h, highlighting the impact of weight status on metabolomic responses and nutrient adaptation [26].

Metabolomic studies have observed various alterations in the plasma metabolome during an oral glucose tolerance test (OGTT). Fatty acids and amino acids have emerged as reliable indicators of metabolic regulation [27]. Specifically, medium-chain acylcarnitines have been associated with insulin resistance [28]. Additionally, metabolites such as free fatty acids, acylcarnitines, bile acids, and lysophosphatidylcholines have been recognized as discriminatory biomarkers during an OGTT [29]. These studies have created a new test for impaired glucose tolerance (IGT) based on all metabolites [30]. Moreover, metabolic phenotyping has been utilized to distinguish different responders to an OGTT, with unique metabolic reactions being correlated with an “at risk” phenotype [31].

We have previously shown that consuming different macronutrients (glucose, protein, and lipids) at a caloric intake of 300 kcal can cause significant changes in plasma glucose and insulin levels in healthy individuals. More precisely, the intake of glucose and protein led to a marked rise in postprandial plasma insulin levels at 1–2 h and 1–3 h, respectively. On the other hand, the consumption of lipids did not significantly affect plasma insulin levels [32]. The regulation of insulin secretion involves a complex interplay of metabolites and signaling pathways triggered by nutrient intake. Glucose ingestion initiates anaplerosis and cataplerosis in pancreatic beta-cells, producing citrate, malate, and malonyl-CoA, which are pivotal in insulin secretion [33].

Furthermore, mitochondrial phosphoenolpyruvate carboxykinase (PEPCK-M) and mitochondrial guanosine triphosphate (mtGTP) are crucial in glucose-stimulated insulin secretion [34]. On the other hand, lipids activate the G-protein-coupled receptor 40 (GPR40) receptor pathway, enhancing glucose metabolism and insulin secretion but with a delayed effect compared to glucose [35]. Notably, 3′-O-methylguanosine (3′-OMG), an endogenous methylated guanosine, exhibits distinct dynamics following glucose and lipid intake. After glucose ingestion, 3′-OMG levels began to decrease during the first hour, reaching their lowest point at 2 h. By contrast, during lipid ingestion, 3′-OMG levels remained stable in the first hour but increased at the 2 h mark. Guanosine and its derivatives, which are known to regulate insulin sensitivity [34,36], play a role in the mitochondrial GTP (mtGTP) cycle, linking glucose metabolism to insulin secretion through succinyl-CoA synthetase (SCS-GTP) and mitochondrial PEPCK-M [34].

This interplay elucidates the nuanced regulation of circulating insulin levels following human nutrient intake. Glucose-induced 3′-OMG dynamics reflect pathways like anaplerosis, cataplerosis, and the mtGTP cycle, promptly enhancing insulin release. By contrast, lipids trigger a delayed response due to processes like lipolysis and fatty acid oxidation modulating metabolic intermediates that indirectly affect insulin secretion. These distinct metabolic responses underscore the differential effects of glucose and lipids on insulin levels. Moreover, the involvement of nucleotides and metabolites, including 3′-OMG, alongside guanosine derivatives and the mtGTP cycle, emphasizes the complex metabolic regulation in pancreatic beta cells, offering insights into managing insulin sensitivity and related disorders.

The response to glucose intake after a meal is a complex process that involves intricate metabolic interactions. Insulin, which plays a crucial role in regulating nutrient metabolism, has significant effects on various metabolic pathways. One of these effects is the substantial reduction in plasma triglyceride (TG) levels when insulin is infused, indicating its involvement in nutrient partitioning. This reduction is likely mediated by the activation of glutamate dehydrogenase (GDH), which promotes the oxidation of carbohydrates [37]. However, the response to glucose intake is not uniform, as evidenced by the different patterns of TG levels observed after consuming glucose and lipids (Figure 5).

Interestingly, TG(15:0/18:4/18:1) showed a significant decrease at 1 h followed by a sharp increase at 2 h post-glucose intake, contrasting with the pattern observed after lipid ingestion. This fluctuation in TG levels may be linked to insulin secretion timing and its subsequent metabolic effects. Insulin’s ability to control nutrient partitioning through receptor signaling in peripheral organs such as the liver, muscle, adipose tissue, and brain is well established [38].

The decrease in poly-g-D-glutamate levels could be attributed to increased glutamate flux into the tricarboxylic acid (TCA) cycle, facilitated by insulin-mediated enhancement of carbohydrate oxidation [7]. GDH plays a key role in this process, regulating the entry of glutamate into the TCA cycle and influencing α-ketoglutarate formation [37]. Activation of GDH by positive regulators during the postprandial phase supports glutamate flux into the TCA cycle, contributing to metabolic adjustments following nutrient intake [37].

The findings of this investigation provide a more comprehensive understanding of the postprandial metabolomic profile following the consumption of equicaloric amounts of different macronutrients at different time points. However, it is important to acknowledge that certain limitations in this study may warrant further exploration in future research endeavors. Firstly, it is worth noting that the study exclusively focused on individuals who were deemed healthy. Consequently, the implications of metabolomic alterations in individuals with metabolic disorders characterized by insulin resistance, such as diabetes and obesity, were not elucidated. Additionally, the sample sizes of the study groups were relatively small, which may have impacted the generalizability of the results. Furthermore, the majority of participants in each group were male, thereby precluding an investigation into potential gender differences in the postprandial response to the macronutrient challenge [13]. Another limitation of this study is the use of a non-targeted metabolomic approach, which provides relative rather than absolute quantification of metabolites. While this method is powerful in detecting changes in metabolite levels, it does not allow for the determination of absolute concentrations. Therefore, the physiological significance of the dysregulated metabolites remains speculative. To better understand the clinical and biological implications of these findings, future studies should incorporate targeted metabolomics methods that can offer absolute quantification of metabolite concentrations, allowing for a more comprehensive interpretation of metabolic changes in response to dietary challenges.

We acknowledge that a cross-over design using a Latin square approach could have provided additional insight into inter-individual variability and minimized potential confounding factors by allowing participants to serve as their own controls. However, due to the study’s primary objective of comparing distinct macronutrient-induced metabolic responses, we opted for a parallel-group design. Future studies should consider adopting a cross-over design with a Latin square approach to further enhance the robustness of the findings and explore within-subject variability in postprandial responses.

Another limitation is the administration of isolated nutrients, which may not fully reflect typical dietary practices. Meals in real-world scenarios are more complex, containing multiple macronutrients that interact in various ways. While the glucose bolus was used to simulate a high glycemic response, this does not accurately represent a common dietary scenario. Similarly, isolated protein powder and cream do not capture the digestibility and metabolic effects of whole food sources. Future research should prioritize mixed meal studies to enhance the applicability of the findings in practical nutritional contexts.

This study provides valuable insights for the field of postprandial metabolomics by providing a comprehensive comparison of metabolic responses to glucose, protein, and lipid intake. Unlike many previous studies that focused solely on glucose or mixed meals, our study uniquely examines the distinct postprandial metabolomic profiles of isolated macronutrients, offering a clearer understanding of their specific metabolic pathways and impacts. The application of an untargeted metabolomic approach allows for the identification of novel biomarkers and metabolic pathways that could be overlooked in targeted studies. Despite the sample size limitation, our findings align with and expand upon the metabolomic data from similar studies. The parallel-group design remains scientifically sound for our research objectives, and future work could benefit from a cross-over approach to further validate the findings.

Lastly, the study was strengthened by the homogeneity of the recruited subjects in terms of age, health status, and body mass index (BMI), as well as the application of untargeted metabolomic LC/MS analysis, a highly sensitive and comprehensive analytical platform. These factors, combined with our innovative design, contribute meaningfully to the growing body of research on postprandial metabolomics.

## 5. Conclusions

The study identified distinct patterns of dysregulated endogenous metabolites in response to each macronutrient challenge, highlighting the complexity and specificity of the metabolic pathways involved in glucose, protein, and lipid metabolism. A total of 21 dysregulated metabolites were observed after glucose ingestion, compared to 59 and 156 dysregulated metabolites following protein and lipid intake, respectively. Four metabolites—3′-O-methylguanosine, 3-oxotetradecanoic acid, poly-g-D-glutamate, and triglyceride (TG) (15:0/18:4/18:1)—were commonly dysregulated between the glucose and lipid groups. No common dysregulated metabolites were identified between the protein and glucose groups or between the protein and lipid groups. These findings enhance our understanding of the metabolic processes triggered by different macronutrients and provide insights into the biochemical pathways involved in postprandial metabolism, which could be valuable for assessing metabolic health.

## Figures and Tables

**Table 1:** Demographic data and clinical characteristics of the study population.

Group	Gender (M/F)	Age (Years)	BMI (kg/m^2^)	Time	Insulin (µU/mL)	Glucose (mg/dL)
**Glucose** **(N = 7)**	(6/1)	21.7 ± 0.47	21.2 ± 0.87	0 h	4.3 ± 0.23	92.3 ± 1.90
				1 h	42.2 ± 18.80 *	95.0 ± 12.98
				2 h	12.8 ± 1.35 *	75.1 ± 7.88
				3 h	4.1 ± 0.82	72.3 ± 13.05
**Protein** **(N = 10)**	(10/0)	21.4 ± 0.24	22.1 ± 0.47	0 h	7.518 ± 0.812	88.2 ± 1.75
				1 h	28.483 ± 4.048 *	82.8 ± 2.42
				2 h	22.458 ± 3.3 *	80.8 ± 1.71
				3 h	16.722 ± 2.258	81.4 ± 2.15
**Lipid** **(N = 6)**	(6/0)	21.3 ± 0.33	22.4 ± 1.18	0 h	8.3 ± 1.77	90.7 ± 2.77
				1 h	11.2 ± 2.60	73.7 ± 3.22 *
				2 h	6.8 ± 1.45	82.2 ± 4.55
				3 h	5.6 ± 1.58	79.3 ± 3.53

* The asterisks (*) in the table indicate that the values are statistically significant compared to control measurements, with a significance level of *p* < 0.05.

## Data Availability

All the data has been included either in the paper or in Supplementary Table S1.

## Supplementary Materials

The following supporting information can be downloaded at: https://www.mdpi.com/article/10.3390/nu16213783/s1, Table S1: Endogenous metabolites were identified as significantly dysregulated for glucose after applying one-way ANOVA at multiple time points (0, 1, 2, and 3); Table S2: Endogenous metabolites were identified as significantly dysregulated for protein after applying one-way ANOVA at multiple time points (0, 1, 2, and 3); Table S3: Endogenous metabolites were identified as significantly dysregulated for lipid after applying one-way ANOVA at multiple time points (0, 1, 2, and 3).
